# Investigating the Mechanism of Hyperglycemia-Induced Fetal Cardiac Hypertrophy

**DOI:** 10.1371/journal.pone.0139141

**Published:** 2015-09-29

**Authors:** Sha-sha Han, Guang Wang, Ya Jin, Zheng-lai Ma, Wei-jing Jia, Xia Wu, Xiao-yu Wang, Mei-yao He, Xin Cheng, Wei-jing Li, Xuesong Yang, Guo-sheng Liu

**Affiliations:** 1 Department of Pediatrics and Neonatology, Institute of Fetal-Preterm Labor Medicine, The First Affiliated Hospital, Jinan University, Guangzhou, 510630, China; 2 Division of Histology & Embryology, Key Laboratory for Regenerative Medicine of the Ministry of Education, Medical College, Jinan University, Guangzhou, 510632, China; 3 Department of Fetal Medicine, The First Affiliated Hospital, Jinan University, Guangzhou, 510630, China; University of Central Florida, UNITED STATES

## Abstract

Hyperglycemia in diabetic mothers enhances the risk of fetal cardiac hypertrophy during gestation. However, the mechanism of high-glucose-induced cardiac hypertrophy is not largely understood. In this study, we first demonstrated that the incidence rate of cardiac hypertrophy dramatically increased in fetuses of diabetic mothers using color ultrasound examination. In addition, human fetal cardiac hypertrophy was successfully mimicked in a streptozotocin (STZ)-induced diabetes mouse model, in which mouse cardiac hypertrophy was diagnosed using type-M ultrasound and a histological assay. PH3 immunofluorescent staining of mouse fetal hearts and *in vitro*-cultured H9c2 cells indicated that cell proliferation decreased in E18.5, E15.5 and E13.5 mice, and cell apoptosis in H9c2 cells increased in the presence of high glucose in a dose-dependent manner. Next, we found that the individual cardiomyocyte size increased in pre-gestational diabetes mellitus mice and in response to high glucose exposure. Meanwhile, the expression of β-MHC and BMP-10 was up-regulated. Nkx2.5 immunofluorescent staining showed that the expression of Nkx2.5, a crucial cardiac transcription factor, was suppressed in the ventricular septum, left ventricular wall and right ventricular wall of E18.5, E15.5 and E13.5 mouse hearts. However, cardiac hypertrophy did not morphologically occur in E13.5 mouse hearts. In cultured H9c2 cells exposed to high glucose, Nkx2.5 expression decreased, as detected by both immunostaining and western blotting, and the expression of KCNE1 and Cx43 was also restricted. Taken together, alterations in cell size rather than cell proliferation or apoptosis are responsible for hyperglycemia-induced fetal cardiac hypertrophy. The aberrant expression of Nkx2.5 and its regulatory target genes in the presence of high glucose could be a principal component of pathogenesis in the development of fetal cardiac hypertrophy.

## Introduction

Pregnant women with either pre-gestational diabetes mellitus (PGDM) or gestational diabetes mellitus are at high risk (2–5 times higher than non-diabetic pregnancies) of carrying a fetus with congenital anomalies, such as phocomelia, cardiac malformations, macrosomia and central nervous system malformations [1, 2]. Among those congenital malformations induced by hyperglycemia, congenital heart disease [3] and anomalies of the nervous system are prominent [4] because both the cardiovascular and nervous systems start to develop during the early embryonic developmental stage, the period that is vulnerable to external harmful factors. Hyperglycemia is hypothesized to be the most important teratogen affecting cardiovascular formation by generating excess reactive oxygen species (ROS) [5, 6]. These congenital developmental defects might include artery or pulmonary atresia, double-outlet of the right ventricle, tetralogy of Fallot, and fetal cardiomyopathy [7]. Diabetic cardiomyopathy induced predominantly by continuous hyperglycemia manifests as cardiac hypertrophy, and heart failure would occur if serious pathogenesis existed [8]. The excessive production of ROS is one factor that is responsible for the development of diabetic cardiomyopathy [9]. There is no doubt that other factors are also involved in the pathogenesis of diabetic cardiomyopathy, either through participation in the production of ROS or independent of ROS production. However, the exact pathogenesis of hyperglycemia-induced cardiac hypertrophy in a developing fetus still remains controversial. In this study, we investigated the cellular and molecular biological pathogenesis of cardiac hypertrophy using streptozotocin (STZ)-induced diabetic mice and H9c2 cells.

The heart forms from the primitive heart tube, which is previously derived from the fusion of the bilateral endocardial tubes. The primitive heart tube forms a C-loop modeling and further separates into the four chambers to form an adult heart eventually. The major components of the embryonic heart are composed of the cardiac precursor cells, the cardiac neural crest and the pro-epicardium [10]. During cardiogenesis, a higher risk of congenital heart disease is understandable if the migration or differentiation of cardiac precursor cells is abnormal. For example, alteration in the embryological origin of the right and left ventricular myocardium is implicated in congenital heart disease [11]. Another reason that the heart is susceptible to external and internal harmful factors is that heart development itself is a very complicated process. Normal cardiogenesis relies on precise spatiotemporal regulation by heart-development-related genes at different embryonic developmental stages. Fibroblast growth factor signaling is required for the migration of cardiac progenitor cells in the primary heart field of Drosophila and chick [12, 13]. This cardiac precursor specification occurs when these cells reach the anterior lateral plate mesoderm. As an important inducer of myocardium, bone morphogenetic protein 2 (BMP2) expressed by the anterior endoderm plays an important role in cardiac induction in the chick embryo. Moreover, Nkx2.5 and GATA4, along with myocardin and Tbx20, are well-known transcription factors that characterize and regulate the cardiogenic differentiation of cardiac processor cells. In particular, Nkx2.5 and BMP2 are indispensable for cardiogenic induction [14].

Using the diabetes mouse model induced by STZ and the H9c2 cell line, we investigated the cellular and molecular biological mechanism through which high glucose in diabetic pregnancy causes cardiac hypertrophy.

## Materials and Methods

### 1.1 Experimental Animals

The Kunming mice that were used in the current study were obtained from the Laboratory Animal Centre of Sun Yat-sen University (Guangzhou, China). Eight-week-old female mice were used to induce diabetes mellitus by injecting STZ (Sigma, St. Louis, MO, USA; dissolved in 0.01 mol/l citrate buffer, pH 4.5) at 75 mg/kg body weight for three consecutive days. The blood glucose level was measured by the Roche Accu-Chek Aviva Blood Glucose System (Roche, Penzberg, BY, Germany) for 7 days after STZ injection. Diabetes mellitus was characterized as non-fasting blood glucose level exceeding 16 mmol/l [15, 16]. In contrast, before and during pregnancy, euglycemia (4–8 mmol/l) sustained in control mice. Two female mice were housed with one normal male mouse overnight in a cage. The day that the vaginal plugs were observed was designated as embryonic day 0.5 (E0.5). During pregnancy, the blood glucose level was monitored every 6 days. At E13.5, E15.5 and E18.5, the embryos were dissected by caesarean section after the pregnant mice were anaesthetized by intraperitoneally injecting pentobarbital (150 mg/kg body weight). This study was carried out in strict accordance with the recommendations of the Guide for the Care and Use of Laboratory Animals of the National Institutes of Health. The protocol was approved by the Committee on the Ethics of Animal Experiments of the Jinan University. All surgery was performed under pentobarbital anesthesia, and all efforts were made to minimize suffering.

### 1.2 Echocardiography analysis

The clinical study was performed between Jan. 1^st^, 2011 and Dec. 31^st^, 2012 in The First Affiliated Hospital, Jinan University (Guangzhou, China). All of the color ultrasound examinations were performed using the Voluson E8 system (GE Healthcare, Little Chalfont, UK) with transabdominal 4-8-MHz curvilinear transducers. Our study was conducted in compliance with the Declaration of Helsinki. The study protocol was approved by the Ethics Committee of the First Affiliated Hospital of Jinan University (Guangzhou, China) and all participants provided written informed consents. For the *in vivo* study, transthoracic echocardiographic examinations were conducted using the Vevo770^TM^ imaging system (VisualSonics Inc., Toronto, Ontario, Canada) in control and PGDM mice on E18.5. Pregnant female mice were sedated using 1.5% isoflurane and laid supine on a heating pad, with the legs taped to an electrocardiogram electrode for heart rate monitoring at 450–550 beats/min, and their body temperature was maintained at 36 to 38°C. Pre-warmed ultrasound gel was applied on the site that the hairs on the abdomen were removed. Measurements were obtained using M-mode imaging with standard clinical ultrasound imaging planes.

### 1.3 Morphological analysis

To examine whether or not diabetes mellitus altered the morphology of the heart, the embryo hearts were harvested at each assigned time. These embryo hearts or embryos were photographed and then fixed in 4% paraformaldehyde. After dehydrated, the embryos are embedded in paraffin wax and serially sectioned at 4 μm for hematoxylin and eosin (H&E) staining. The sections were photographed using an Olympus LX51 fluorescent microscope (Leica, Wetzlar, Germany) with NIS-Elements F3.2 software. The average size (area) of the cardiomyocytes was determined and divided by the total area of the microscopic field by the total number of cardiomyocytes present, as previously described [17]. A minimum of 5 random images from 5 samples were assayed per group and per assigned time.

### 1.4 Immunofluorescent staining

Immunofluorescent staining was performed on paraffin vertical sections using p-histone H3 (PH3) and Nkx2.5 antibodies. Briefly, the vertical sections were de-waxed in xylene, rehydrated and then heated in a microwave for antigen retrieval before exposure to the primary antibody with citrate buffer (pH = 6.0). Unspecific immunoreactions were blocked using 5% inactivated goat serum in PBS for 30 min at room temperature. The sections were washed in PBS and incubated with rabbit polyclonal PH3 antibody (1:200, Santa, Santa Cruz, CA, USA) or rabbit polyclonal Nkx2.5 antibody (1:200, Abcam, New Territories, HK) overnight at 4°C. Following extensive washing, the sections were incubated in goat anti-rabbit IgG secondary antibody that was conjugated to Alexa Fluor 555 dyes (1:500, Invitrogen, Waltham, MA, USA) for three hours at room temperature in a dark box. After immunostaining, all of the sections were counterstained with DAPI (1:1000, Invitrogen, Waltham, MA, USA) for 30 min at room temperature.

### 1.5 Cell culture

The H9c2 rat cardiac myoblast cell line was obtained from ATCC (American Type Culture Collection, CLR-1446, USA). The cells were cultured in a humidified incubator with 5% CO_2_ at 37°C in six-well plates (1×10^6^ cells/ml) containing DMEM (Gibco, Gaithersburg, MD, USA) that was supplemented with 10% fetal bovine serum (Gibco, Gaithersburg, MD, USA) and exposed to various concentrations of glucose (5.5 mmol/l, 25 mmol/l, 50 mmol/l D-glucose, Sigma, St. Louis, MO, USA); 5.5 mmol/l D-glucose was used as a control. The cells were photographed using an inverted fluorescence microscope (Nikon, Tokyo, Japan) with NIS-Elements F3.2 software. After 72 hours of incubation, immunofluorescent staining with Alexa Fluor 594 phalloidin (F-actin, 1:1000, Invitrogen, Waltham, MA, USA) and anti-Nkx2.5 (1:100, Abcam, New Territories, HK) was performed on the incubated H9c2 cells. A minimum of 5 images were assayed per treatment group.

### 1.6 3-(4,5-dimethylthiazol-2-yl)-2,5-diphenyltetrazolium bromide (MTT) assay

The cell viability was assessed using a modified MTT assay. Briefly, 10 μl of MTT solution (5 mg/ml in PBS) was added to 96-well plates and incubated continually for 4 hours at 37°C. The MTT solution was then removed, and formazan dye was dissolved in dimethyl sulfoxide (DMSO, Sigma-Aldrich, MO, USA) for 10 min by shaking. The absorbance values were measured at 570 nm using a Bio-Rad Model 450 Microplate Reader (Bio-Rad, Hercules, CA, USA). The cell viability was indirectly established by a ratio of the absorbance value of 25 mmol/l and 50 mmol/l D-glucose-treated cells relative to the control (5.5 mmol/l). The final results were determined by analyzing three independent experiments.

### 1.7 Quantitation of apoptotic cells

The double staining of Annexin V-FITC (BD, Franklin Lakes, NJ, USA) and propidium iodide (PI) double staining was used to identify and quantify the apoptotic cells that were present in the cell cultures. Briefly, the cultured cells were immediately analyzed by a FACSAria flow cytometer (BD, Franklin Lakes, NJ, USA). The acquired data were evaluated using FACSDiva V4.1.2 software (BD, Franklin Lakes, NJ, USA).

### 1.8 RNA isolation and RT-PCR

Total RNA was isolated from H9c2 cells using the E.Z.N.A.® Total RNA Kit (OMEGA, Norcross, GA, USA) according to the manufacturer's instructions. Reverse transcription to synthesize cDNA was performed using the PrimeScript^TM^ RT Reagent Kit with gDNA Eraser (Takara, Shiga, Japan). PCR amplification of the cDNA was performed using specific primers for rat KCNE1 (5’-TGTGGCAGGAAACAGATGAG-3’ and 5’-GGGTGAAGAAGCCGAAGAA-3’; 132 bp 56°C), Cx43 (5'-GTCTCACCTTTGTGCCTTCC-3' and 5'-GCTCACCTCCCTGATGCTAA-3'; 146 bp 56°C), BMP10 (5'-TGTCCATCCCTCACCACGAAGA-3' and 5’-TCCGTTGATACCAAGACCAGCA-3’; 175 bp 56°C), β-MHC (5’-CCCCTACGATTATGCGTTCTTC-3' and 5’-TCCTCCCTCTGCTTCTGTTTGA-3'; 195 bp 56°C) and β-actin (5’-CAACCTTCTTGCAGCTCCTCCGTC-3’ and 5’-TCTGACCCATACCCACCATCACACC-3’; 200 bp 56°C). PCR was performed in a Bio-Rad S1000^TM^ Thermal cycler (Bio-Rad, Hercules, CA, USA). cDNAs were amplified for 40 cycles. One round of amplification was performed at 95°C for 5 sec, at 56°C for 30 sec and at 72°C for 30 sec (Takara, Shiga, Japan). The PCR products (20 μl) were resolved on 2% agarose gels (Sigma, St. Louis, MO, USA) in 1× TAE buffer (0.04 M Tris acetate and 0.001 M EDTA), and GeneGreen Nucleic Acid Dye (TIANGEN, Beijng, China). The reaction products were visualized using a transilluminator (SYNGENE, Cambridge, UK) and a computer-assisted gel documentation system (SYNGENE, Cambridge, UK).

### 1.9 Western blot

Western blotting was performed in accordance with a standard procedure using a polyclonal antibody that specifically recognizes Nkx2.5. The collected hearts from E13.5, E15.5 and E18.5 developing embryos were frozen in liquid nitrogen and kept at -80°C. The protein from the hearts or H9c2 cells was isolated from tissue homogenates or cell lysate using a radio-immuno-precipitation assay (Sigma, St. Louis, MO, USA) buffer that was supplemented with protease and phosphatase inhibitors. The protein concentrations were quantified using the BCA assay. The extracted protein was separated by 10% sodium dodecyl sulfate polyacrylamide gel electrophoresis and transferred onto a polyvinylidene difluoride membrane (Millipore, Billerica, MA, USA). The membrane was blocked with 5% nonfat milk and then incubated with anti-Nkx2.5 antibody (1:1000, Abcam, New Territories, HK) in TBS buffer at 4°C overnight. The loading control was β-actin antibody (1:3000, Santa, Santa Cruz, CA, USA). After incubation with the secondary antibody HRP goat anti-rabbit IgG (1:3000, EarthOx, Millbrae, CA, USA), the blots were developed with SuperSignal^TM^ West Femto Chemiluminescent Substrate (ThermoFisher, Waltham, MA, USA), and the Gel Doc™ XR+ System (Bio-Rad, Hercules, CA, USA) and Quantity One software were used to capture the chemiluminescent signals.

### 1.10. Data analysis

Data analyses and the construction of statistical charts were performed using the GraphPad Prism 5 software package (GraphPad Software, La Jolla, CA, USA). The results were presented as a mean value ($\bar{x}\pm s$). The data were analyzed using ANOVA or *t* tests, which were employed to establish whether there was any difference between the control and experimental data. P<0.05 was considered significantly significant.

## Results

### 2.1 Diabetes in human pregnancy caused cardiac hypertrophy of the developing fetus

**Fig 1:**
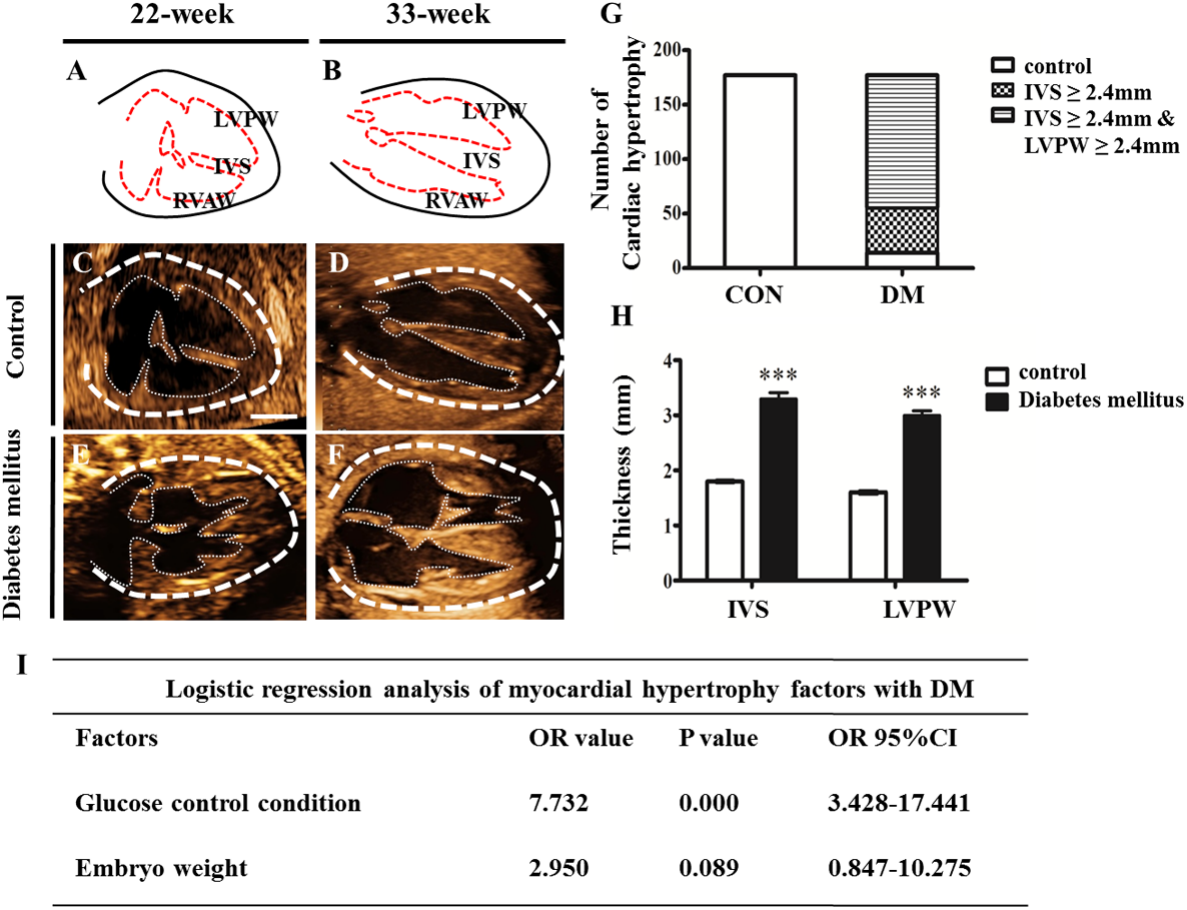
Pregnancy associated with diabetes caused cardiac hypertrophy of human fetus. **A-B**: The schematic drawings for the structures of human fetal hearts at the 22nd week (A) and the 33rd week (B) of gestation. **C-D**: The representative images of heart color ultrasound from normal pregnancy (control) at the 22nd week (C) and 33rd week (D) of gestation, respectively. **E-F**: The representative images of heart color ultrasound from pregnancy associated with diabetes mellitus (DM) at the 22nd week (E) and 33rd week (F) of gestation, respectively. **G**: Bar charts showing the occurrence of a variety of hypertrophies in the control and DM groups. Cardiac hypertrophy is divided into two types: single IVS≥2.4 mm and both IVS and LVPW≥2.4 mm. **H**: Bar charts showing the average thickness (mm) of IVS and LVPW in heart color ultrasound from the control and DM groups. **I**: The logistic regression analysis of myocardial hypertrophy factors with DM. ***P<0.001 vs control. N = 177. Scale bars: 5 mm in C-F.

To determine the effect of diabetes on the fetal heart development, we examined 177 fetal hearts with diabetic mothers, including those suffering from gestational diabetes mellitus and from pre-existing diabetes, using color ultrasound. There is no clinical uniform diagnostic standard for fetal cardiac hypertrophy. However, fetal cardiac hypertrophy could occur if the interventricular septum (IVS) is greater than two standard deviations. The reports [18] showed that the value of normal full-term fetal IVS and left ventricular posterior wall (LVPW) was 2.4 mm in a color ultrasound. Therefore, we used the value 2.4 mm as the standard for the examination of fetal hearts using color ultrasound. From the results of clinical color ultrasound examination (Fig 1C–1F), we can see that the percentage of only IVS ≥ 2.4 mm in the diabetes mellitus group was 23% (n = 41 in total 177), and the percentage of both IVS and LVPW ≥ 2.4 mm in the diabetes mellitus group was 69% (n = 122 in total 177) (Fig 1G). The average thickness of IVS in the diabetes mellitus group (3.3±1.5 mm, n = 177) was much greater than the one in the control group (1.8±0.3 mm [18]; P<0.001); similarly, the average thickness of LVPW in the diabetes mellitus group (3.0±1.1 mm, n = 177) was also much greater than the one in the control group (1.6±0.4 mm [18]; P<0.001) (Fig 1H). The data from the color ultrasound indicate that cardiac hypertrophy occurred in pregnancy with diabetes mellitus. Meanwhile, a logistic regression analysis of myocardial hypertrophy factors with diabetes mellitus showed that the odds ratio (OR) value of the glucose control condition was 7.732, indicating a more than seven times greater risk of cardiac hypertrophy in pregnancy with diabetes mellitus (Fig 1I).

### 2.2 Human fetal cardiac hypertrophy could be mimicked in the mice with PGDM

**Fig 2:**
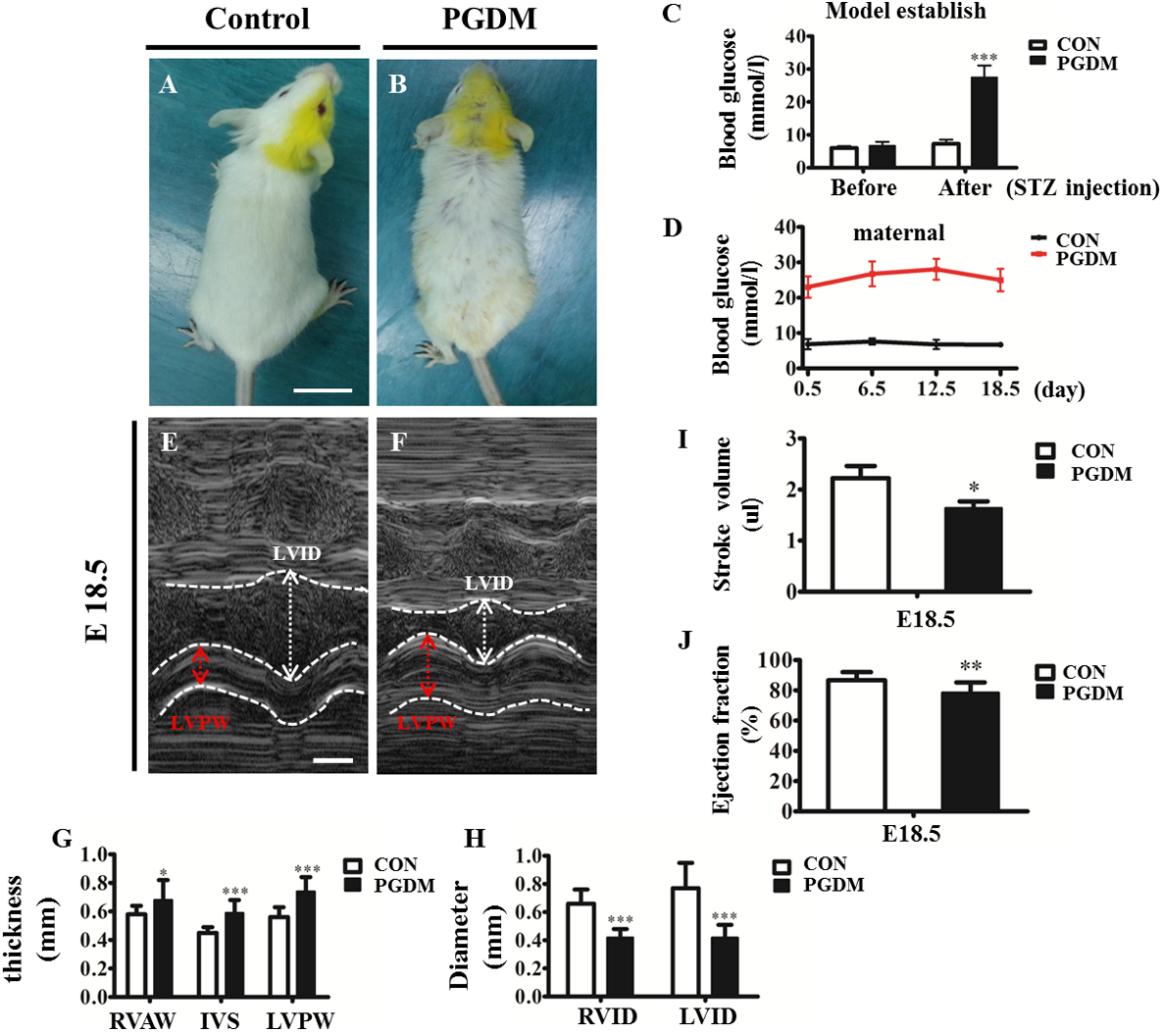
Cardiac hypertrophy occurred in the embryos of STZ-induced diabetic mice. **A-B**: The representative mice from the control (A) and PGDM groups (B). **C**: The bar charts showing the levels of blood glucose in the control and PGDM mice before and after STZ injection. **D**: The graph showing the levels of blood glucose in the control and PGDM mice from E0.5 to E18.5 after fertilization. **E-F**: The representative images from M-type ultrasound of fetal mouse hearts in the control (E) and PDGM groups (F). The red arrows indicate the thickness of the LVPW, and the white arrows indicate the left ventricular internal diameter. **G**: The bar charts showing the thicknesses of the cardiac RVAW, IVS and LVPW in the control and PGDM mice at E18.5 after fertilization. **H**: The bar charts showing the diameters of the ventricular cavities in the control and PGDM mice at E18.5 after fertilization. **I**: The bar charts showing the stroke volume in the control and PGDM mice at E18.5 after fertilization. **J**: The bar charts showing the ejection fraction in the control and PGDM mice at E18.5 after fertilization. *P<0.05, **P<0.01, ***P<0.001 vs control. Scale bars: 1 cm in A-B and 500 μm in E-F.

**Fig 3:**
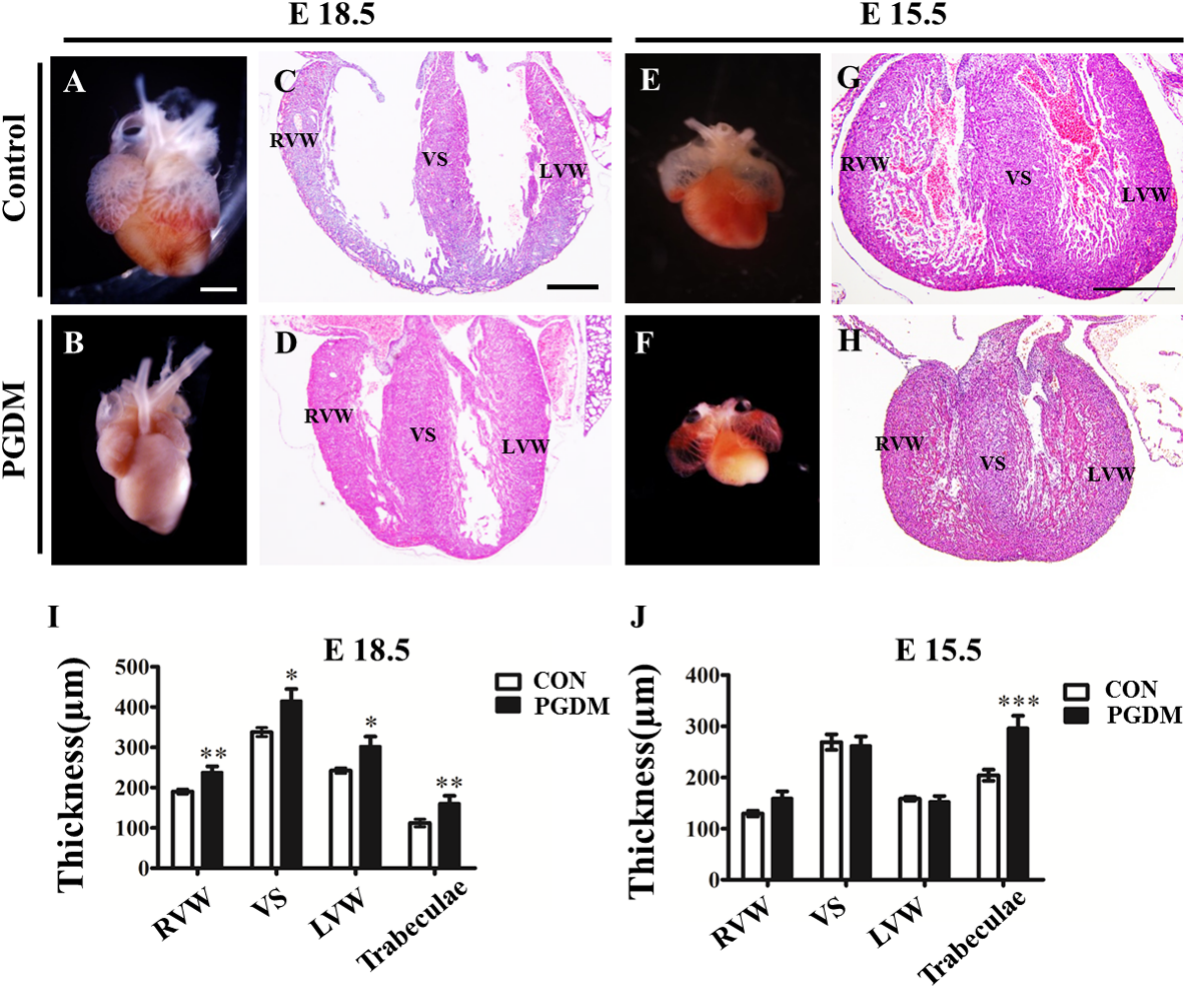
Cardiac hypertrophy occurred in the prenatal mice with PGDM. **A-B**: The representative E18.5 mouse hearts from the control (A) and PGDM (B) groups. **C-D**: The H&E stained vertical sections of the E18.5 mouse hearts from the control (C) and PGDM (D) groups. **E-F**: The representative E15.5 mouse hearts from the control (E) and PGDM (F) groups. **G-H**: The H&E stained vertical sections of the E15.5 mouse hearts from the control (G) and PGDM (H) groups. **I**: The bar chart showing the thicknesses of the RVW, VS, LVW and trabeculae in the E18.5 mice from the control and PGDM groups. **J**: The bar chart showing the thicknesses of the RVW, VS, LVW and trabeculae in the E15.5 mice from the control and PGDM groups. *P<0.05, **P<0.01, ***P<0.001 vs control. Scale bars: 1000 μm in A-B, E-F and 400 μm in C-D, G-H.

To investigate the mechanism of diabetes mellitus-induced cardiac hypertrophy, we established a mouse model of PGDM through the intraperitoneal injection of STZ as previously described [15]. The mouse blood glucose level was monitored to verify success in creating a mouse model of diabetes mellitus. One week after the administration of STZ, the blood glucose level in the diabetes mellitus group increased from 6.70±1.19 mmol/l (n = 24, before STZ administration) to 27.45±3.64 mmol/l (n = 24, after STZ administration), which was significantly different from the blood glucose level in the control group (7.28±1.26 mmol/l, n = 24; P<0.001) (Fig 2C). This result suggests that a maternal mouse model of diabetes mellitus was successfully established. The maternal blood glucose level was maintained at the high level (more than 20 mmol/l) for the entire gestational period in the diabetes mellitus mice; meanwhile, there was no increase in the blood glucose in the control group (Fig 2D). Therefore, we can ensure that any phenotype in the mouse model of diabetes mellitus was associated with high levels of glucose in the blood. The symptoms in the pregnant mice with diabetes mellitus included polydipsia, polyphagia, polyuria and weight loss, the typical characteristics for diabetes mellitus, along with loss of hair gloss, as shown in Fig 2A and 2B. To determine whether or not the pregnant maternal mice had diabetes mellitus, we monitored the heart development of E18.5 fetal mice using a type-M ultrasound. The results showed that the thickness of the right ventricular anterior wall (RVAW) in the diabetes mellitus group (0.68±0.14 mm, n = 10) was significantly greater than that in the control group (0.58±0.06 mm, n = 10; P<0.05); the thickness of IVS in the diabetes mellitus group (0.59±0.09 mm, n = 10) was significantly greater than that in the control group (0.45±0.04 mm, n = 10; P<0.001); and the thickness of LVPW in the diabetes mellitus group (0.74±0.10 mm, n = 10) was significantly greater than that in the control group (0.56±0.07 mm, n = 10; P<0.001) (Fig 2E–2G). In addition to the thickness of the cardiac wall, we also measured the diameter of the cardiac cavities in the absence/presence of high glucose using a type-M ultrasound. The results indicated that the right ventricular internal diameter (RVID) in the diabetes mellitus group (0.42±0.06 mm, n = 10) was less than that in the control group (0.66±0.10 mm, n = 10; P<0.001); the left ventricular internal diameter (LVID) in the diabetes mellitus group (0.42±0.06 mm, n = 10) was less than that in the control group (0.77±0.18 mm, n = 10; P<0.001) (Fig 2H). From the vertical sections, we determined that the hypertrophic heart and cardiac cavity shrank in E18.5 and E15.5 PDGM mouse hearts compared to those of the control group at the same developmental stage (Fig 3). Carefully measuring the cardiac walls in different regions showed that the right ventricular wall (RVW) in the E18.5 PGDM group (238.00±94.19 μm, n = 8) was thicker than that in the control group (190.04±39.46 μm, n = 8; P<0.01); the ventricular septum (VS) in the E18.5 PGDM group (415.70±183.35 μm, n = 8) was thicker than that in the control group (337.87±70.30 μm, n = 8; P<0.05); the left ventricular wall (LVW) in the E18.5 PGDM group (320.90±151.44 μm, n = 8) was thicker than that in the control group (242.36±42.46 μm, n = 8; P<0.05); and the trabeculae in the E18.5 PGDM group (161.09±117.52 μm, n = 8) was thicker than that in the control group (112.26±61.56 μm, n = 8; P<0.01) (Fig 3I). Interestingly, we did not find significant difference in the thickness of the RVW, VS and LVW between the PGDM and control groups except for thicker trabeculae in the PGDM group (296.51±149.52 μm, n = 8) than in the control group (204.12±68.71 μm, n = 8; P<0.001) at E15.5 (Fig 3J).

The examination of the type-M ultrasound also identified cardiac functional indicators, such as stroke volume and ejection fraction. In the mice with diabetes mellitus, the stroke volume (1.64±0.13 μl, n = 10) was less than that in the control group (2.22±0.24 μl, n = 10; P<0.05) (Fig 2I), and the ejection fraction (78.42±6.79%, n = 10) was also less than that in the control group (86.65±5.39%, n = 10; P<0.01) (Fig 2J), indicating that the normal physiological functions of the fetal mouse were restricted in the presence of high glucose despite cardiac hypertrophy.

### 2.3 PGDM inhibited cell proliferation and promoted cell apoptosis

**Fig 4:**
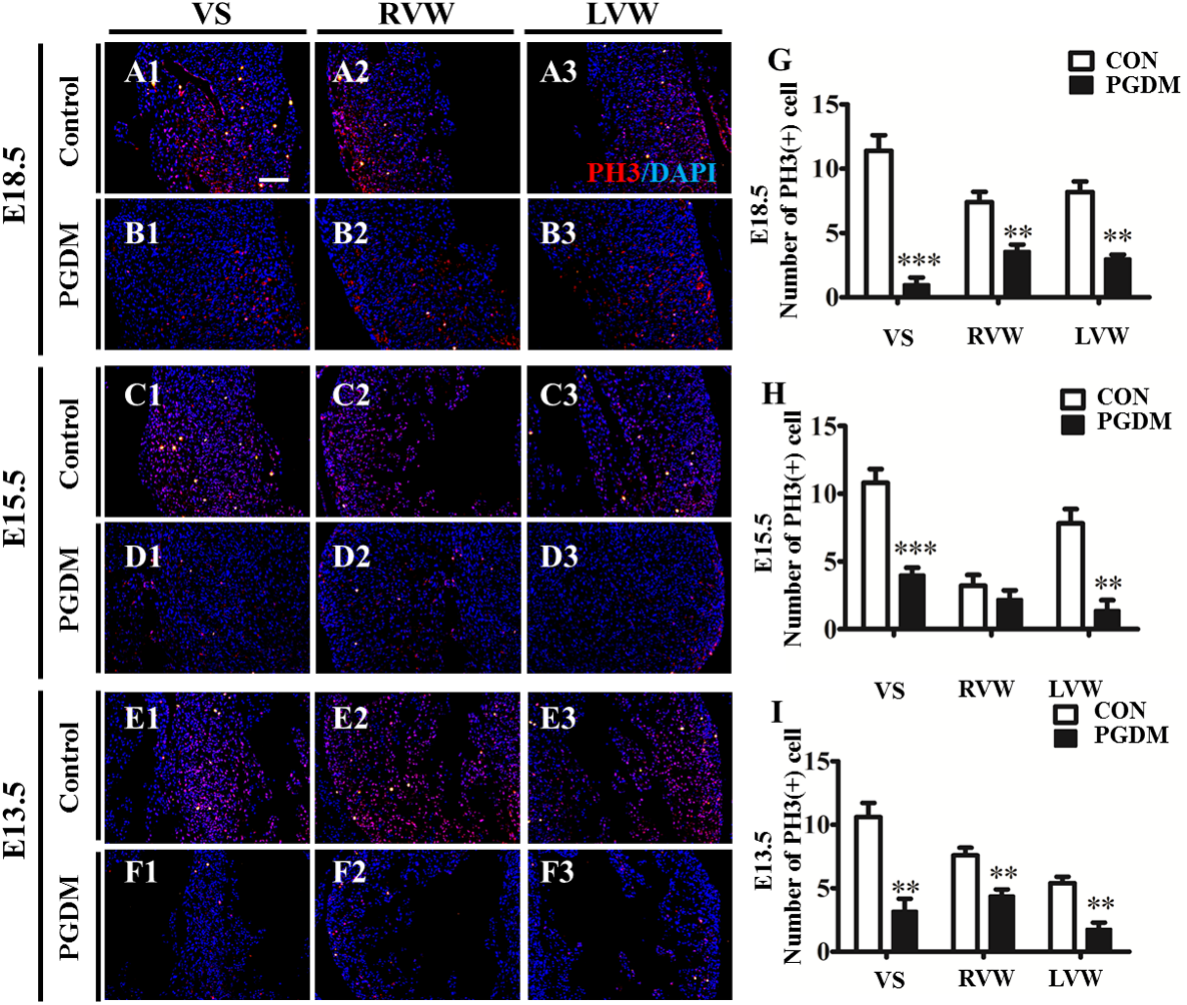
PGDM inhibited cell proliferation in prenatal mouse hearts. PH3 immunofluorescent staining (red) was performed on the vertical sections of the prenatal mouse hearts. All of the sections were counterstained with DAPI (blue). **A1-A3/B1-B3**: The vertical sections of E18.5 mouse hearts from the control cardiac VS (A1), RVW (A2), and LVW (A3) and PGDM cardiac VS (B1), RVW (B2), and LVW (B3). **C1-C3/D1-D3**: The vertical sections of E15.5 mouse hearts from the control cardiac VS (C1), RVW (C2), and LVW (C3) and PGDM cardiac VS (D1), RVW (D2), and LVW (D3). **E1-E3/F1-F3**: The vertical sections of E13.5 mouse hearts from the control cardiac VS (E1), RVW (E2), and LVW (E3) and PGDM cardiac VS (F1), RVW (F2), and LVW (F3). **G**: The bar charts showing the numbers of PH3^+^ cells in the VS, RVW and LVW of the E18.5 control and PGDM mice. **H**: The bar charts showing the numbers of PH3^+^ cells in the VS, RVW and LVW of the E15.5 control and PGDM mice. **I**: The bar charts showing the numbers of PH3^+^ cells in the VS, RVW and LVW of the E13.5 control and PGDM mice. **P<0.01, ***P<0.001 vs control. Scale bars: 100 μm in A1-F3.

Because the fetal heart is in the process of development, it seems likely that cardiac hypertrophy might be due to the alteration of cell proliferation and/or apoptosis in the developing heart. First, we examined whether cell proliferation was responsible for PGDM-induced cardiac hypertrophy using immunofluorescent staining of PH3, a marker of cell proliferation. The PH3^+^ cell numbers in the VS, RVW and LVW of the E18.5, E15.5 and E13.5 PGDM groups were lower than their corresponding controls (Fig 4A1–4F3). In E18.5 mice, the PH3^+^ cell number in the VS, RVW and LVW was 1.0±1.22, 3.6±1.14 and 3.0±0.71, respectively, which was significantly lower than in the control group (11.4±2.7, 7.4±1.82 and 8.2±1.79, n = 5; P<0.01) (Fig 4G). In E15.5 mice, the PH3^+^ cell number in the VS, RVW and LVW was 4.0±1.22, 3.2±1.79 and 1.2±1.3, respectively, which was significantly lower than that of the control group (10.8±2.28, 3.2±1.79 and 7.8±2.39, n = 5; P<0.01 for IVS and LVW, P>0.05 for RVW) (Fig 4H). In E13.5 mice, the PH3^+^ cell number in the VS, RVW and LVW was 3.2±2.16, 4.4±1.44 and 1.8±1.10, respectively, which was significantly lower than that of the control group (10.6±2.51, 7.6±1.34, 5.4±1.14, n = 5; P<0.01) (Fig 4I). This result indicates that PGDM inhibited cell proliferation in nearly every region of the developing heart.

**Fig 5:**
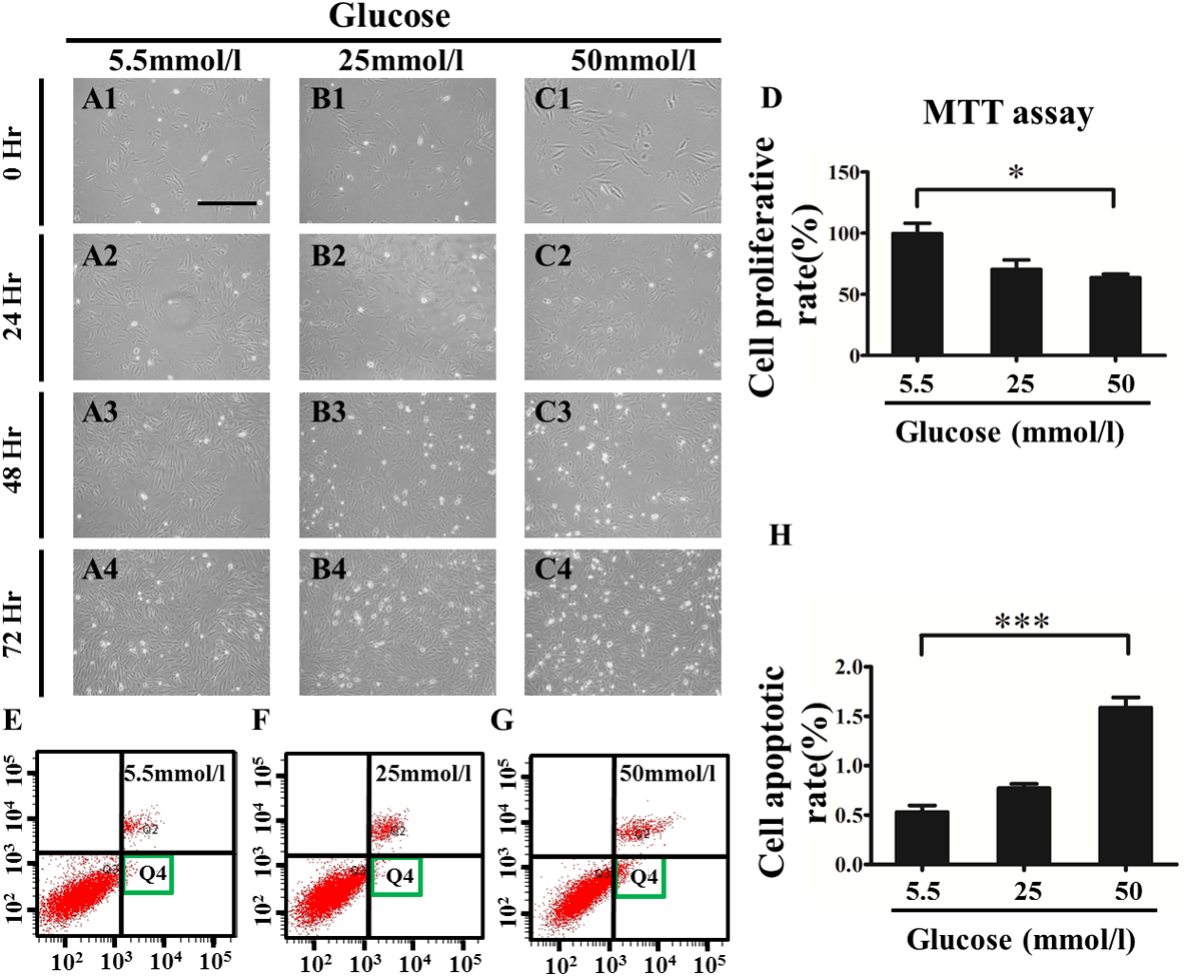
Exposure to high glucose restricted cell proliferation and promoted the cell apoptosis of cardiomyocytes in vitro. The H9c2 cells were treated with different concentrations of glucose for 72 hours. **A1-A4**: Representative images of 5.5-mmol/l-glucose-treated cells that were cultured for 0 (A1), 24 (A2), 48 (A3) and 72 (A4) hours. **B1-B4**: Representative images of 25-mmol/l-glucose-treated cells that were cultured for 0 (B1), 24 (B2), 48 (B3) and 72 (B4) hours. **C1-C4**: Representative images of 50-mmol/l-glucose-treated cells that were cultured for 0 (C1), 24 (C2), 48 (C3) and 72 (C4) hours. **D**: The bar chart showing the relative percentage of cell proliferations in the presence of various concentrations of glucose. **E-G**: The flow cytometry assay showing the distributions of apoptosis cells in the presence of 5.5 mmol/l (E), 25 mmol/l (F) and 50 mmol/l (G) glucose. **H**: The bar chart showing the percentage of apoptosis cells in the presence of various concentrations of glucose. *P<0.05, ***P<0.001 vs control. Scale bars: 200 μm in A1-C4.

To further investigate cell proliferation and apoptosis, H9c2 cells [19], a myoblast cell line, were employed to evaluate the effect of high glucose *in vitro*. The H9c2 cells were administered with various concentrations of glucose, including 5.5 mmol/l, 25 mmol/l and 50 mmol/l [19], for 72 hours before being harvested for further analysis. The incubation time of 72 hours was chosen because there was no significant change in the cell viability in the MTT assay at either 24 or 48 hours of incubation, and a distinct alteration appeared at 72 hours of incubation (Fig 5D). To investigate cell apoptosis in the presence of high glucose, flow cytometry with double staining of Annexin V/PI was employed using the Annexin V-FITC kit, and demonstrated that the cell population in zone Q4 in the 50-mmol/l glucose group (1.59±0.18%, n = 3) was significantly higher than that in the 5.5-mmol/l group (control) (0.53±0.12%, n = 3; P<0.001) (Fig 5E–5G and 5H). Taken together, the high glucose exposure in PGDM could inhibit cell proliferation and promote cell apoptosis in developing hearts.

### 2.4 PGDM increased the size of individual cardiomyocytes in the prenatal heart

**Fig 6:**
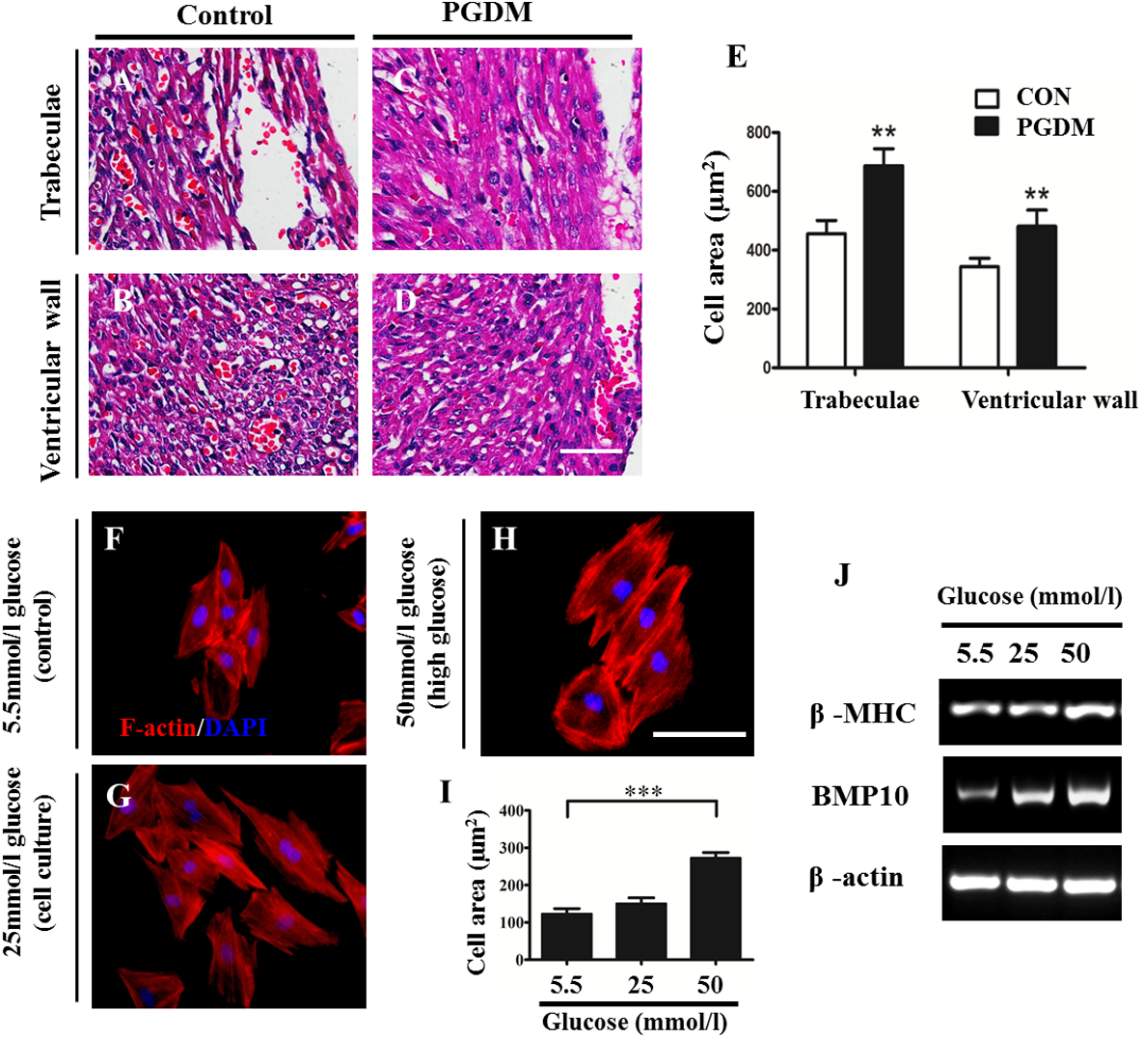
The size of individual cardiomyocytes increased in the embryos of PGDM mice. **A-B:** The representative images of H&E-stained vertical sections of the trabeculae (A) and ventricular wall (B) of the E18.5 control mice. **C-D:** The representative images of H&E-stained vertical sections of the trabeculae (C) and ventricular wall (D) of the E18.5 PGDM mice. **E**: Bar charts showing the individual cell area (μm^2^) in the trabeculae and ventricular wall of the E18.5 control and PGDM mice. **F-H:** The representative merged images of F-actin immunofluorescence and DAPI staining for incubated H9c2 cells after exposure to 5.5 mmol/l (F), 25 mmol/l (G) and 50 mmol/l (H) glucose for 72 hours. **I**: Bar charts showing the individual H9c2 cell area (μm^2^) after exposure to 5.5 mmol/l, 25 mmol/l and 50 mmol/l glucose for 72 hours. **J**: RT-PCR data showing the mRNA levels of β-MHC and BMP10 in the incubated H9c2 cells after exposure to 5.5 mmol/l, 25 mmol/l and 50 mmol/l glucose for 72 hours. **P<0.01, ***P<0.001 vs control. Scale bars: 100 μm in A-D and 100 μm in F-H.

The alteration of the individual cardiomyocyte size is another possibility that could lead to cardiac hypertrophy after excluding cell proliferation and apoptosis as mentioned above. Vertical sections of the trabeculae and ventricular wall of control and PGDM mouse hearts were produced and stained with H&E. The average size of the cardiomyocytes in the representative vertical sections was calculated by dividing the total area that the cells occupied by the total cell number in the histological sections (Fig 6A–6D). Using this simple method, we found that the cardiomyocytes significantly increased in size in the PGDM trabeculae (687.99±164.49 μm^2^, n = 8; P<0.01) and ventricular wall (481.02±134.79 μm^2^, n = 8; P<0.01) compared to those in the control group (455.89±128.48 μm^2^, n = 8; 394±61.89 μm^2^, n = 8) (Fig 6E). In addition, H9c2 cells (the cardiac myoblast cell line) were exposed to different concentrations of glucose (5.5 mmol/l, 25 mmol/l and 50 mmol/l) before the immunofluorescent staining of F-actin to outline the cell contours (Fig 6F–6H). Again, we found that the H9c2 cells significantly increased in size in a dose-dependent manner after exposure to glucose (123.26±31.38 μm^2^ in 5.5 mmol/l and 272.71±25.81 μm^2^ in 50 mmol/l. n = 5; P<0.01) (Fig 6I). Furthermore, the expression of β-MHC and BMP10, which are genes related to size increase, was up-regulated in a dose-dependent manner (5.5 mmol/l, 25 mmol/l and 50 mmol/l) (Fig 6J). These data suggest that the individual size increase could partially be responsible for the cardiac hypertrophy induced by PGDM.

### 2.5 The down-regulation of Nkx2.5 and downstream genes in the developing hearts of mice with PGDM

**Fig 7:**
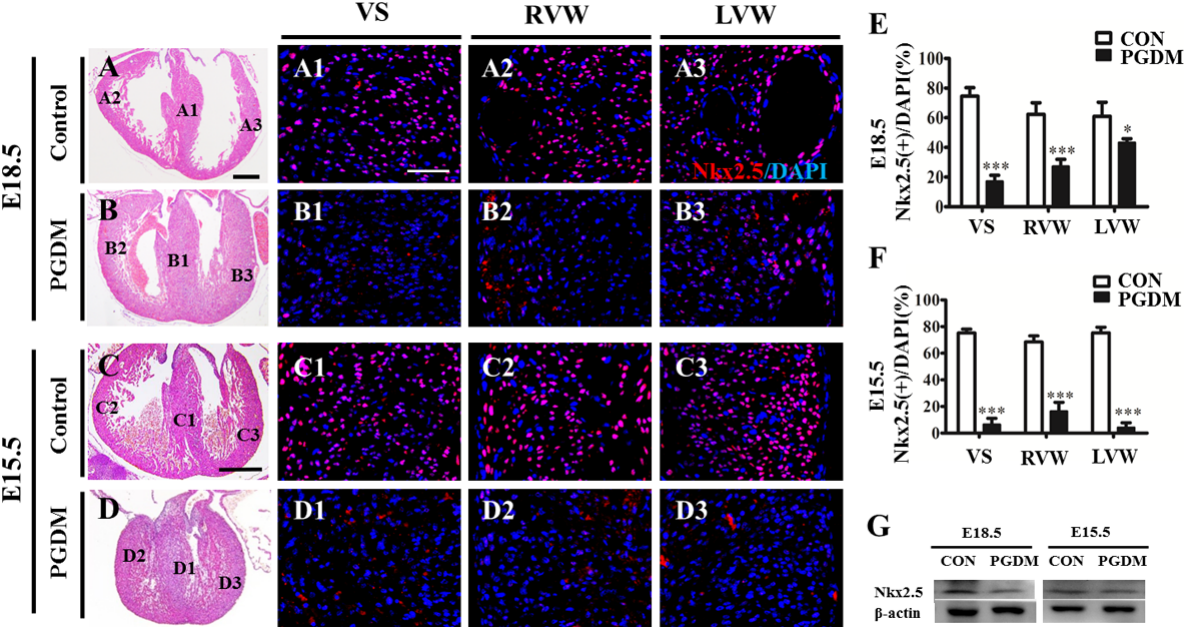
Nkx2.5 expression was suppressed in E18.5 and E15.5 PGDM mice. Nkx2.5 immunofluorescent staining (red) was performed on the vertical sections of the prenatal mouse hearts. All of the sections were counterstained with DAPI (blue). **A-D:** The representative images of H&E-stained vertical sections from the E18.5 control (A), E18.5 PGDM (B), E15.5 control (C) and E15.5 PDGM (D) mice. **A1-A3/B1-B3**: The vertical sections of the E18.5 mouse hearts from control cardiac VS (A1), RVW (A2), and LVW (A3) and PGDM cardiac VS (B1), RVW (B2), and LVW (B3). **C1-C3/D1-D3**: The vertical sections of E15.5 mouse hearts from control cardiac VS (C1), RVW (C2), and LVW (C3) and PGDM cardiac VS (D1), RVW (D2), and LVW (D3). **E**: Bar charts showing the percentage of Nkx2.5^+^ cells/DAPI cells in the VS, RVW and LVW of the E18.5 control and PGDM mice. **F**: Bar charts showing the percentage of Nkx2.5^+^ cells/DAPI cells in the VS, RVW and LVW of the E15.5 control and PGDM mice. **G**: Western blotting data showing Nkx2.5 expression at the protein level in E18.5 or E15.5 control and PGDM mice. *P<0.05, ***P<0.001 vs control. Scale bars: 400 μm in A-D and 100 μm in A1-D3.

Nkx2.5 is indispensable for the differentiation of cardiac cardiomyocytes due to its role in regulating the expression of several essential transcription factors during heart formation [20, 21]. Therefore, we determined the expression of Nkx2.5 in the VS, RVW and LVW from the E18.5 and E15.5 control or PGDM mouse hearts. From Nkx2.5 immunofluorescent staining, we found that the numbers of Nkx2.5^+^ cells in the PGDM group were much lower than those in the control group (details following), in either the E18.5 or E15.5 mouse hearts (Fig 7A1–7D3). In the E18.5 mouse hearts, the number of Nkx2.5^+^ cells in the VS of the PGDM group (17.15±4.03%, n = 5) was significantly lower than that in the control group (74.56±5.74%, n = 5; P<0.001); the number of Nkx2.5^+^ cells in RVW of the PGDM group (27.21±4.72%, n = 5) was significantly lower than that in the control group (62.27±7.86%, n = 5; P<0.001); and the number of Nkx2.5^+^ cells in LVW of the PGDM group (43.32±2.59%, n = 5) was significantly lower than that in the control group (60.93±9.52%, n = 5; P<0.05) (Fig 7E). The numbers of Nkx2.5^+^ cells in the VS, RVW and LVW of the E15.5 PGDM mouse hearts (6.50±4.60%, 16.42±6.67% and 4.26±3.53%, respectively; n = 5) were also significantly lower than those in the control group (75.22±2.88%, 68.42±4.62% and 75.25±4.30%, respectively; n = 5; P<0.001) (Fig 7F). The western blotting data also showed reduced Nkx2.5 expression at the protein level in the E18.5 and E15.5 PGDM mouse hearts compared with that of the controls (Fig 7G).

**Fig 8:**
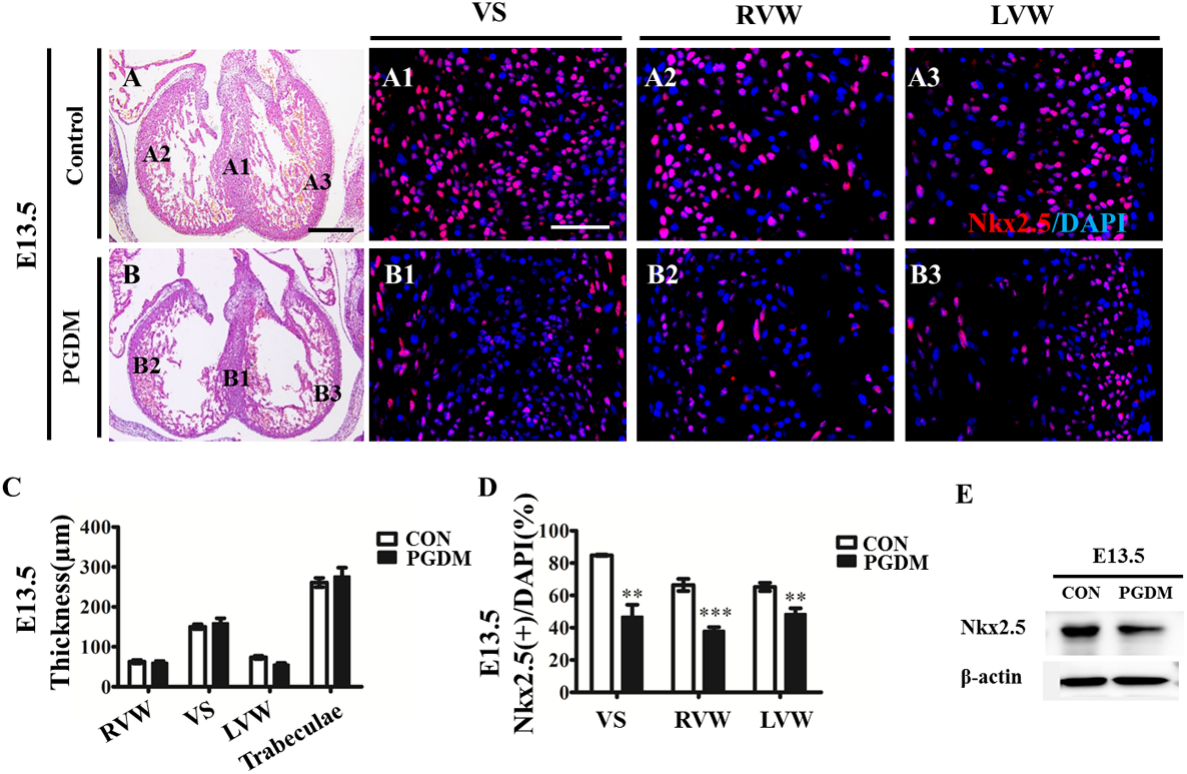
Nkx2.5 was down-regulated, but no cardiac hypertrophy appeared in E13.5 PGDM mice. Nkx2.5 immunofluorescent staining (red) was performed on the vertical sections of the prenatal mouse hearts. All of the sections were counterstained with DAPI (blue). **A-B:** The representative images of H&E-stained vertical sections from the E13.5 control (A) and PDGM (B) mice. **A1-A3/B1-B3**: The vertical sections of the E13.5 mouse hearts from the control cardiac VS (A1), RVW (A2), and LVW (A3) and PGDM cardiac VS (B1), RVW (B2), and LVW (B3). **C**: Bar charts showing the thicknesses of the cardiac VS, RVW, LVW and trabeculae of the E13.5 control and PGDM mice. **D**: Bar charts showing the percentages of Nkx2.5^+^ cells/DAPI^+^ cells in the VS, RVW and LVW of E13.5 control and PGDM mice. **E**: Western blotting data showing Nkx2.5 expression at the protein level in the E13.5 control and PGDM mice. **P<0.01, ***P<0.001 vs control. Scale bars: 400 μm in A-B and 100 μm in A1-B3.

Interestingly, the expression of Nkx2.5 in the VS, RVW and LVW of the E13.5 PGDM mouse hearts (46.86±16.44%, 38.10±5.01% and 48.58±7.81%, respectively; n = 5) was significantly inhibited compared to that of the controls (84.78±0.88%, 66.49±8.37% and 65.24±5.62%, respectively; n = 5; P<0.01) (Fig 8A1–8B3 and 8D) and E18.5 and E15.5 mice. The western blotting data showed a reduction of the Nkx2.5 expression at the protein level as well in the E13.5 PGDM mouse hearts compared to that of the control group (Fig 8E). However, cardiac hypertrophy did not appear at the E13.5 developmental stage, as the measured results indicated no significant difference in the thicknesses of the RVW, VS, LVW and trabeculae between the PGDM and control groups (Fig 8C). This result might suggest that morphological alteration occurs later than the change in the expression of Nkx2.5, the key gene for heart formation.

**Fig 9:**
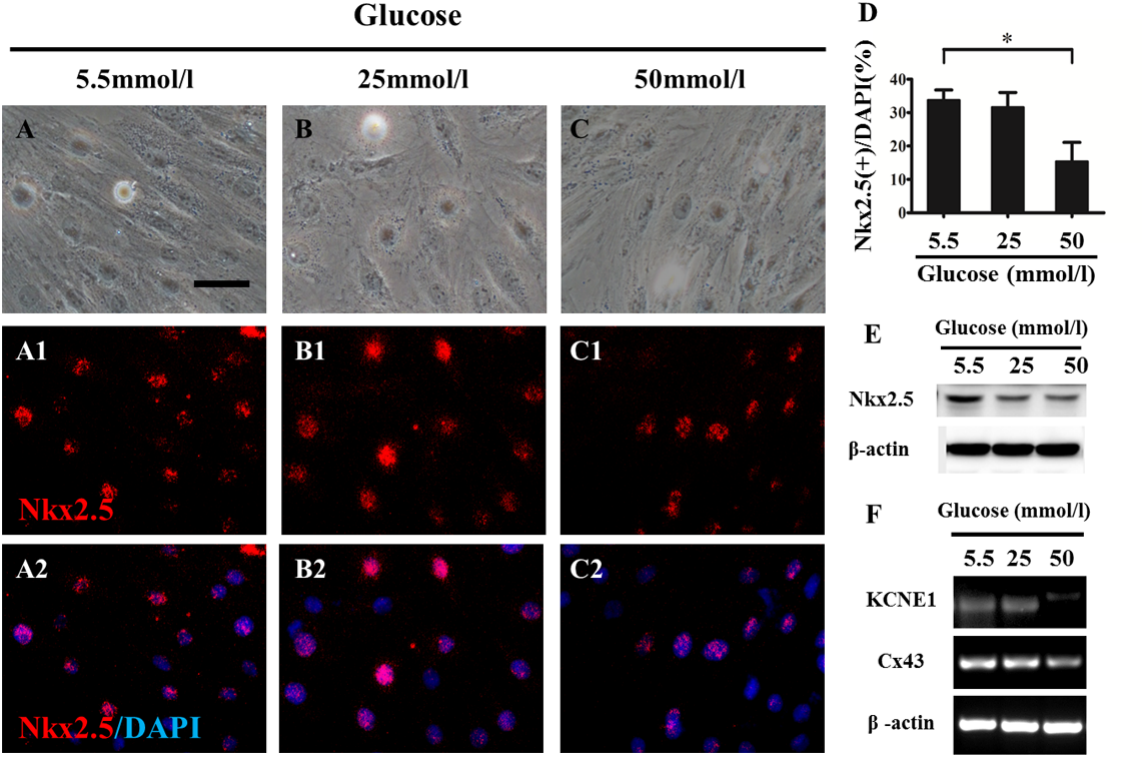
Exposure to high glucose suppressed Nkx2.5 and its downstream gene expressions in cultured cardiomyocytes. The H9c2 cells were incubated *in vitro* with various concentrations of glucose for 72 hours. Nkx2.5 immunofluorescent staining (red) was performed on the cultured cells. All of the cultured cells were counterstained with DAPI (blue). **A-C**: The representative bright-field images of the cells from the 5.5-mmol/l (A), 25-mmol/l (B) and 50-mmol/l (C) groups at 72 hours of incubation. **A1-C1**: The representative Nkx2.5 immunofluorescent staining images from the 5.5-mmol/l (A1), 25-mmol/l (B1) and 50-mmol/l (C1) groups. **A2-C2**: A1-C1 + DAPI counterstain. **D**: The bar charts showing the percentages of Nkx2.5^+^ cells/DAPI^+^ cells in the 5.5-mmol/l, 25-mmol/l and 50-mmol/l groups. **E**: Western blot data showing Nkx2.5 expression at the protein level in the 5.5-mmol/l, 25-mmol/l and 50-mmol/l groups. **F**: RT-PCR data showing the mRNA level of KCNE1 and Cx43 in the 5.5-mmol/l, 25-mmol/l and 50-mmol/l groups. *P<0.05 vs control. Scale bars: 100 μm in A-C, A1-C2.

H9c2 cells that were exposed to a variety of concentrations of glucose *in vitro* were used to determine the effect of the expression of Nkx2.5 and its downstream genes in regulating heart formation (Fig 9A–9C). Nkx2.5 immunofluorescent staining showed that the percentage of Nkx2.5^+^ cell number/total cell number (DAPI^+^ cells) in the 50-mmol/l group (15.40±11.36%, n = 4) was significantly less than that in the 5.5-mmol/l (control) group (33.79±5.96%, n = 4; P<0.05), suggesting that high glucose (50 mmol/l) exposure suppressed the expression of Nkx2.5 compared to that of the control (5.5 mmol/l). Furthermore, western blotting data also indicated the same inhibitive tendency for Nkx2.5 expression at the protein level (Fig 9E). Meanwhile, we detected the expressions of KCNE1 and Cx43, the genes downstream of Nkx2.5, and the RT-PCR data showed that these genes were down-regulated simultaneously (Fig 9F), indicating that the expression of KCNE1 and Cx43 was affected by the down-regulation of Nkx2.5 induced by the high glucose level in PGDM.

## Discussion

There are reports that the collective enlargement of cardiomyocytes is responsible for hypertrophic cardiomyopathy, which might gradually lead to a variety of cardiovascular dysfunctions, such as depressed left ventricular ejection fraction, arrhythmic sudden death, atrial fibrillation and cardiac failure [22–26]. This hypertrophic cardiomyopathy is pathologically different from reactive cardiac hypertrophy, which occurs in response to an extrinsic increase in the cardiac workload caused by hypertension, aortic stenosis and myocardial infarction. Although excess ROS generation is an important pathogenesis, there is no conclusive evidence that demonstrates a link between oxidation stress and cardiac hypertrophy [27]. In addition, many cellular and molecular mechanisms are involved in cardiac hypertrophy, and much less is known about cardiac hypertrophy in the presence of hyperglycemia in the developing fetus. Therefore, it is essential to further investigate the pathogenesis of cardiac hypertrophy induced by hyperglycemia during embryo development, which was the focus of this study.

In our clinical color ultrasound data of fetal hearts during pregnancy with diabetes mellitus, fetal cardiac hypertrophy occurred in most of the pregnancies with diabetes mellitus when the number distributions of embryonic hearts was analyzed according to the thickness of IVS and LVPW. A logistic regression analysis of myocardial hypertrophy factors also indicated that high glucose during pregnancies with diabetes mellitus contributed much more than other factors, such as embryonic weight. There is no doubt that this analysis further verifies the correlation between the high glucose induced by diabetes mellitus and cardiac hypertrophy during gestation. Our observation generally agrees with the report by Amen et al., in which increased cardiac septal thickness could be found in the newborn infants of diabetic mothers despite glycemic control during pregnancy [28]. Next, we successfully established a mouse animal model of diabetes using STZ administration for three consecutive days, significantly increasing the thicknesses of the RVAW, IVS and LVPW of the E18.5 mouse hearts in the presence of high glucose levels in diabetic mouse mothers. However, cardiac dysfunction occurred because both the stroke volume and the cardiac ejection fraction dramatically decreased in STZ-induced mouse fetuses. Meanwhile, cardiac hypertrophy in the fetuses of STZ-induced mice was also histologically confirmed by the corresponding heart vertical sections. These results indicate that a fetal mouse model of cardiac hypertrophy has been successfully established.

In the adult heart, cardiomyocytes are incapable of dividing; therefore, the only way that the heart can accommodate an increased workload is by undergoing compensatory hypertrophy. In contrast, cardiomyocytes in the developing embryonic heart are capable of active division to proliferate and undergo apoptosis. Therefore, we first determined whether cell proliferation was responsible for hyperglycemia-induced cardiac hypertrophy. PH3 could be a good indicator of cell proliferation for interested cells. The numbers of PH3^+^ cardiomyocytes in the VS, RVW and LVW decreased in the E18.5, E15.5 and E13.5 PGDM mice compared to the control group. Moreover, cell proliferation was reduced in H9c2 cells that were cultured *in vitro* [19] and exposed to high glucose. Therefore, reduced cell proliferation could not contribute to cardiac hypertrophy induced by hyperglycemia. Next, the flow cytometry data indicated that the H9c2 cell apoptosis increased in the presence of high glucose, suggesting that the increased cell apoptosis induced by high glucose could not cause cardiac hypotrophy. Therefore, we explored another possibility, the alteration of individual cell size, which probably caused cardiac hypertrophy induced by hyperglycemia in the developing fetal hearts. This speculation was verified by comparing the calculated individual cell area of H&E-stained cardiomyocytes in the PGDM group with that of the control group. Likewise, a similar result was obtained from the *in vitro*-cultured H9c2 cells that were exposed to high glucose; that is, high glucose exposure could lead to increased individual cardiomyocyte size. Except for the cell size, we also discovered that embryonic cardiac hypertrophy was accompanied by increased protein synthesis. Myosin is the main protein constituent that present in hypertrophic cells [29, 30]. We demonstrated that embryonic cardiomyocytes increased myosin synthesis and BMP10 expression [31] (which is related to cardiomyocyte size; for example, BMP10 is responsible for a major component of ventricular muscle defects[32]) in response to high glucose administration in a dose-dependent manner in H9c2 cells. Thus, the experimental results from both *in vivo* and *in vitro* indicate that the increased cardiomyocyte size induced by hyperglycemia was partially responsible for the phenotype of cardiac hypertrophy in the developing hearts.

Nkx2.5 plays an indispensable role in cardiogenesis, and abnormal morphogenesis or/and heart dysfunction occur in the prenatal period and newborn stage when Nkx2.5 is knocked out [20, 33, 34]. In this study, we demonstrated that the expression of Nkx2.5 in the VS, RVW and LVW of the E18.5 and E15.5 PGDM mouse hearts was dramatically suppressed compared to that of the control group, which was again confirmed by western blotting data. Interestingly, we did not morphologically detect cardiac hypertrophy in the E13.5 PGDM mice, although the reduced Nkx2.5 expression was detectable using immunostaining and western blotting assays. The time difference between Nkx2.5 expression and cardiac hypertrophy also verifies that abnormal gene expression occurs before paramorphia, although the causal relationship is unknown. Likewise, the inhibitive effect of high glucose on Nkx2.5 expression by cardiomyocytes was confirmed in cultured H9c2 cells *in vitro*, in which the expression of KCNE1 and Cx43, the downstream target genes of Nkx2.5 [35], was also repressed by high glucose administration. This result provides additional evidence that Nkx2.5 is absolutely indispensable for heart formation because it acts as a cardiac transcription factor to control cardiac gene expression during cardiogenesis and in the process of adaptation to external environments. One could suggest that the mutations of Nkx2.5 are coupled with human congenital heart diseases and heart dysplasia in animal models [32, 36], as we observed here the correlation between the aberrant expression of Nkx2.5 and its downstream genes and cardiac hypertrophy during the prenatal period.

In addition to Nkx2.5, the GATA family and myocyte enhancer factor 2 (MEF2) are also recognized cardiac transcription factors in vertebrate cardiogenesis [36, 37]. Histone acetylation is involved in regulating cardiac transcription factors, for example, O-GlcNAcylation is important in hyperglycemia-induced cardiac hypertrophy through ERK1/2 and cyclin D2 [8]. In addition, the accumulating evidence indicates that excess ROS production is associated with the development of diabetic cardiomyopathy [9], which was also investigated in our previous study [17].

## Conclusion

**Fig 10:**
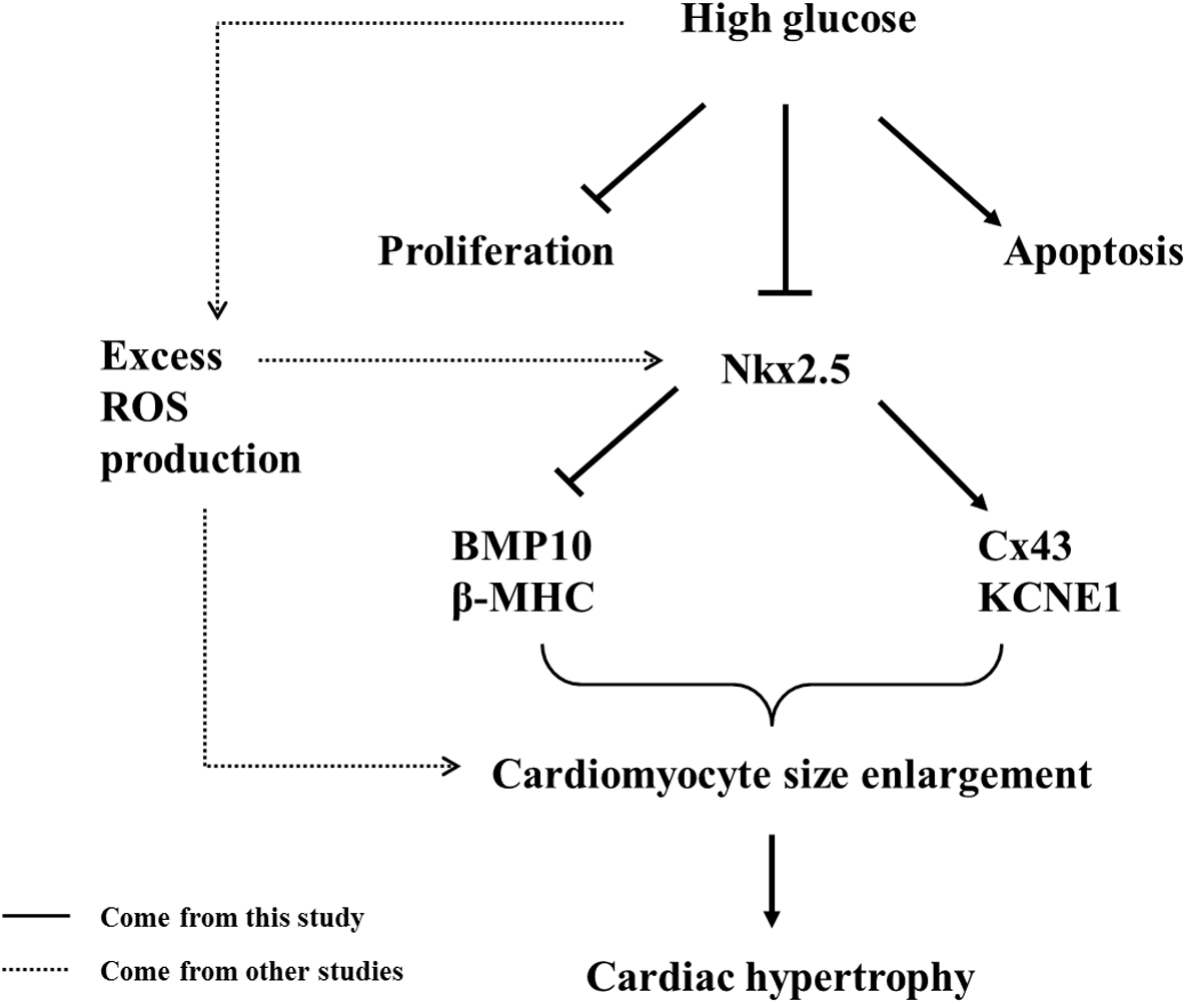
A proposed model illustrating how high glucose in PGDM leads to cardiac hypertrophy during cardiogenesis.

The aim of this study was to investigate the mechanism of the development of hyperglycemia-induced cardiac hypertrophy during cardiogenesis. In summary, we demonstrated that cardiac hypertrophy clinically appeared in the fetal hearts of diabetic mothers using color ultrasound. This cardiac hypertrophy could be mimicked in STZ-induced mice. In the presence of blood high glucose, embryonic cardiomyocyte cell proliferation was inhibited, and cell apoptosis was promoted, which could not contribute to cardiac hypertrophy. High glucose-enhanced cardiomyocyte size might at least partially be responsible for the cardiac hypertrophy. Meanwhile, the expression of the cardiac transcription factor Nkx2.5 was dramatically suppressed, which affected BMP10 and β-MHC (cell size-related) expression and Cx43 and KCNE1 expression (downstream genes). These factors, including excess ROS production, lead to prenatal cardiac hypertrophy (Fig 10). Of course, more precise cellular and molecular biological experiments will be required to provide molecular insight into the mechanisms of normal heart development and fetal cardiac hypertrophy.

## Supporting Information

S1 Fig TUNEL staining on the vertical sections of prenatal mouse hearts. TUNEL immunohistochemical staining (brown) was performed on the vertical sections of the prenatal mouse hearts. All of the sections were counterstained with hematoxylin (blue). A1-A3/B1-B3: The vertical sections of E18.5 mouse hearts from the control cardiac VS (A1), RVW (A2), and LVW (A3) and PGDM cardiac VS (B1), RVW (B2), and LVW (B3). C1-C3/D1-D3: The vertical sections of E15.5 mouse hearts from the control cardiac VS (C1), RVW (C2), and LVW (C3) and PGDM cardiac VS (D1), RVW (D2), and LVW (D3). E1-E3/F1-F3: The vertical sections of E13.5 mouse hearts from the control cardiac VS (E1), RVW (E2), and LVW (E3) and PGDM cardiac VS (F1), RVW (F2), and LVW (F3). G: The bar charts showing the numbers of TUNEL+ cells in the VS, RVW and LVW of the E18.5 control and PGDM mice. H: The bar charts showing the numbers of TUNEL+ cells in the VS, RVW and LVW of the E15.5 control and PGDM mice. I: The bar charts showing the numbers of TUNEL+ cells in the VS, RVW and LVW of the E13.5 control and PGDM mice. *P<0.05, **P<0.01 vs control. Scale bars: 100 μm in A1-F3.
(TIF)

S2 Fig Wheat germ agglutinin (WGA) staining on the vertical sections of E18.5 prenatal mouse hearts. A-B: The representative images for WGA-stained vertical sections of the trabeculae from the control (A) and PGDM (B) groups. C-D: The representative images for WGA-stained vertical sections of the ventricular walls from the control (C) and PGDM (D) groups. E: The bar charts showing the cross-sectional area of mouse myocardiocytes in the trabeculae and ventricular walls of the E18.5 control and PGDM mice. All of the sections were counterstained with DAPI (blue). **P<0.01 vs control. Scale bars: 50 μm in A-D.
(TIF)

## Abbreviations

CON: control
H&E: hematoxylin and eosin
IVS: interventricular septum
LVPW: left ventricular posterior wall
LVW: left ventricular wall
OR: odds ratio
PGDM: pre-gestational diabetes mellitus
RVAW: right ventricular anterior wall
RVW: right ventricular wall
ROS: reactive oxygen species
STZ: streptozotocin
VS: ventricular septum
