# Supplementary figures and images for: Investigating the Mechanism of Hyperglycemia-Induced Fetal Cardiac Hypertrophy
Source: PLoS One. 2015 Sep 29;10(9):e0139141. doi: 10.1371/journal.pone.0139141 (PMC4587747; doi:10.1371/journal.pone.0139141)

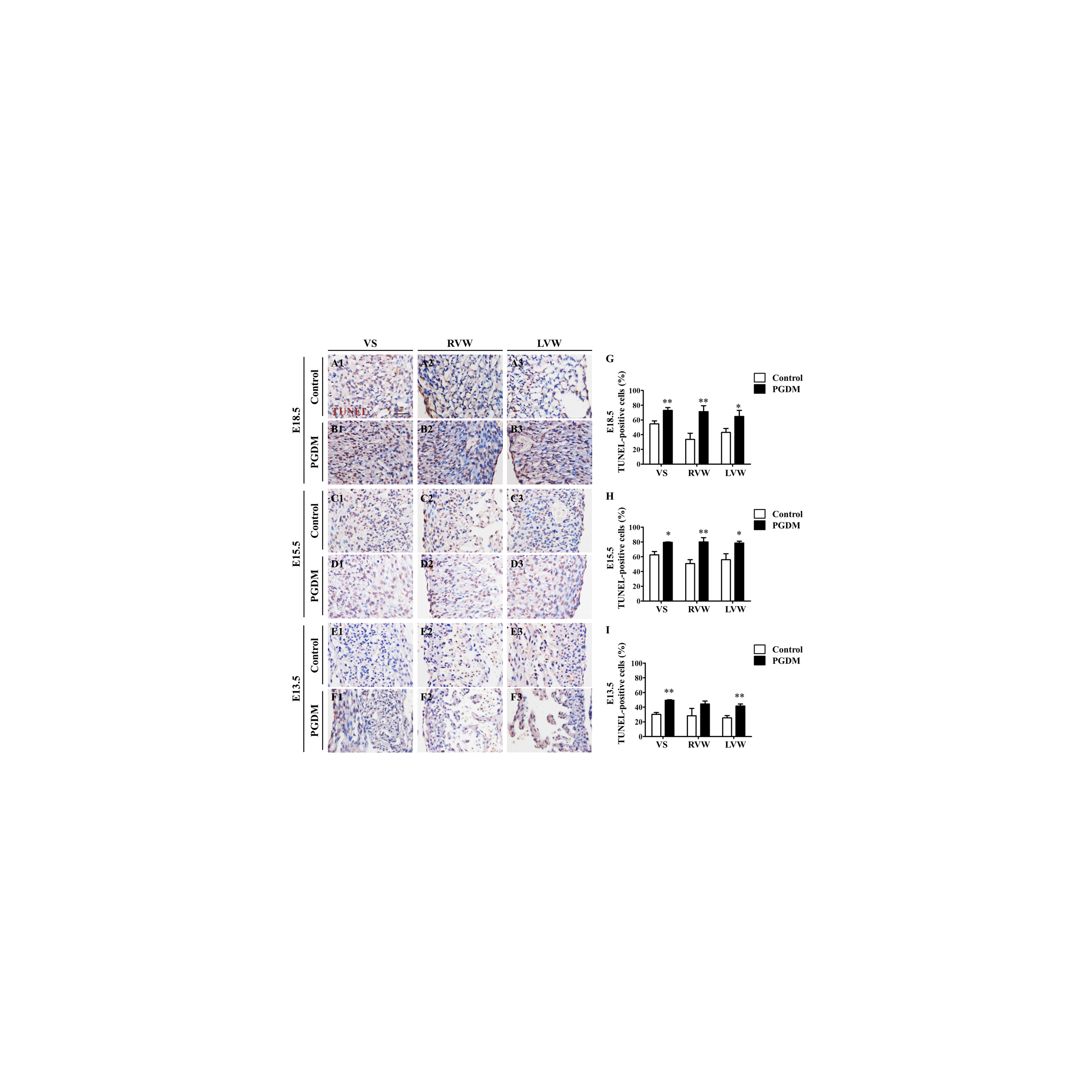

Supplement: S1 Fig — TUNEL immunohistochemical staining (brown) was performed on the vertical sections of the prenatal mouse hearts. All of the sections were counterstained with hematoxylin (blue). A1-A3/B1-B3: The vertical sections of E18.5 mouse hearts from the control cardiac VS (A1), RVW (A2), and LVW (A3) and PGDM cardiac VS (B1), RVW (B2), and LVW (B3). C1-C3/D1-D3: The vertical sections of E15.5 mouse hearts from the control cardiac VS (C1), RVW (C2), and LVW (C3) and PGDM cardiac VS (D1), RVW (D2), and LVW (D3). E1-E3/F1-F3: The vertical sections of E13.5 mouse hearts from the control cardiac VS (E1), RVW (E2), and LVW (E3) and PGDM cardiac VS (F1), RVW (F2), and LVW (F3). G: The bar charts showing the numbers of TUNEL+ cells in the VS, RVW and LVW of the E18.5 control and PGDM mice. H: The bar charts showing the numbers of TUNEL+ cells in the VS, RVW and LVW of the E15.5 control and PGDM mice. I: The bar charts showing the numbers of TUNEL+ cells in the VS, RVW and LVW of the E13.5 control and PGDM mice. *P<0.05, **P<0.01 vs control. Scale bars: 100 μm in A1-F3. (TIF) [file pone.0139141.s001.tif]

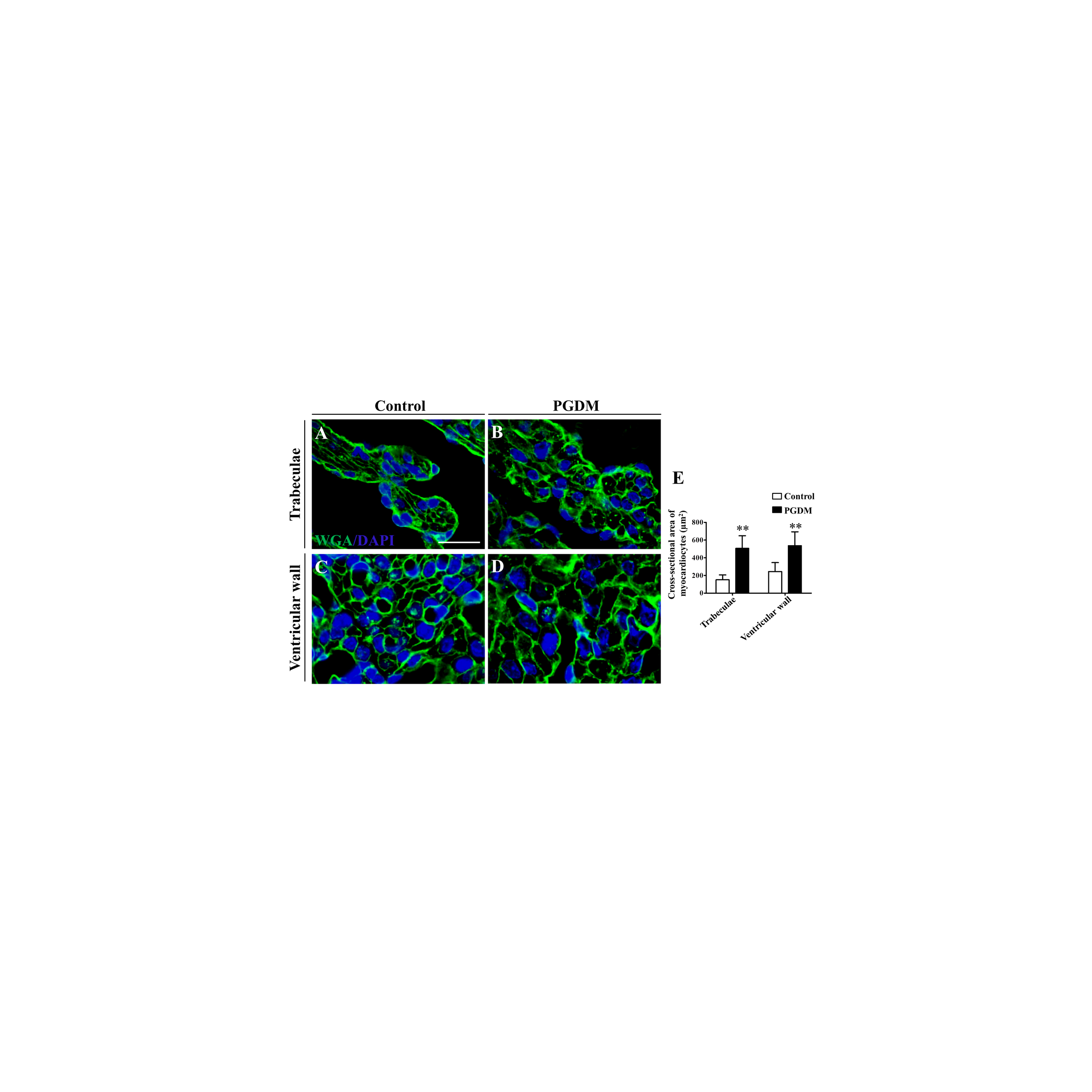

Supplement: S2 Fig — A-B: The representative images for WGA-stained vertical sections of the trabeculae from the control (A) and PGDM (B) groups. C-D: The representative images for WGA-stained vertical sections of the ventricular walls from the control (C) and PGDM (D) groups. E: The bar charts showing the cross-sectional area of mouse myocardiocytes in the trabeculae and ventricular walls of the E18.5 control and PGDM mice. All of the sections were counterstained with DAPI (blue). **P<0.01 vs control. Scale bars: 50 μm in A-D. (TIF) [file pone.0139141.s002.tif]
